# Analysis of Carrier Transport at Zn_1−x_Sn_x_O_y_/Absorber Interface in Sb_2_(S,Se)_3_ Solar Cells

**DOI:** 10.3390/ma17133214

**Published:** 2024-07-01

**Authors:** Junhui Lin, Zhijie Xu, Yingying Guo, Chong Chen, Xiaofang Zhao, Xuefang Chen, Juguang Hu, Guangxing Liang

**Affiliations:** 1 International School of Microelectronics, Dongguan University of Technology, Dongguan 523000, China; 13669872773@163.com (J.L.); 13266001102@163.com (Z.X.); 15322196361@163.com (Y.G.); chong810910@163.com (C.C.); 2 School of Computer Science and Technology, Dongguan University of Technology, Dongguan 523000, China; chenxf@dgut.edu.cn; 3 Shenzhen Key Laboratory of Advanced Thin Films and Applications, Key Laboratory of Optoelectronic Devices and Systems of Ministry of Education and Guangdong Province, State Key Laboratory of Radio Frequency Heterogeneous Integration, College of Physics and Optoelectronic Engineering, Shenzhen University, Shenzhen 518060, China

**Keywords:** ZTO layer, Sb_2_(S,Se)_3_ devices, recombination, Afors-het software

## Abstract

This work explores the effect of a Zn_1−x_Sn_x_O_y_ (ZTO) layer as a potential replacement for CdS in Sb_2_(S,Se)_3_ devices. Through the use of Afors-het software v2.5, it was determined that the ZTO/Sb_2_(S,Se)_3_ interface exhibits a lower conduction band offset (CBO) value of 0.34 eV compared to the CdS/Sb_2_(S,Se)_3_ interface. Lower photo-generated carrier recombination can be obtained at the interface of the ZTO/Sb_2_(S,Se)_3_ heterojunction. In addition, the valence band offset (VBO) value at the ZTO/Sb_2_(S,Se)_3_ interface increases to 1.55 eV. The ZTO layer increases the efficiency of the device from 7.56% to 11.45%. To further investigate the beneficial effect of the ZTO layer on the efficiency of the device, this goal has been achieved by five methods: changing the S content of the absorber, changing the thickness of the absorber, changing the carrier concentration of ZTO, using various Sn/(Zn+Sn) ratios in ZTO, and altering the thickness of the ZTO layer. When the S content in Sb_2_(S,Se)_3_ is around 60% and the carrier concentration is about 10^18^ cm^−3^, the efficiency is optimal. The optimal thickness of the Sb_2_(S,Se)_3_ absorber layer is 260 nm. A ZTO/Sb_2_(S,Se)_3_ interface with a Sn/(Zn+Sn) ratio of 0.18 exhibits a better CBO value. It is also found that a ZTO thickness of 20 nm is needed for the best efficiency.

## 1. Introduction

In recent years, Sb_2_(S,Se)_3_ has been regarded as a potential semiconductor thin film among new-type photovoltaic materials due to its excellent stability, ideal band gap, and suitable absorption coefficient [1,2,3,4]. Sb_2_(S,Se)_3_ is deposited using different technologies, such as vapor transport deposition, rapid thermal evaporation, pulsed laser deposition, hydrothermal methods, spin-coating, and chemical bath deposition [5,6,7,8,9,10]. In 2019, Tang’s group fabricated Sb_2_(S,Se)_3_ devices using rapid thermal evaporation and vapor transport deposition. The best efficiency of the devices was less than 7% [5]. Chen et al. prepared an Sb_2_(S,Se)_3_ device with an efficiency of 7.05% by pulsed laser deposition in 2020 [7]. Recently, solution synthesis processes have also contributed to the deposition of Sb_2_(S,Se)_3_ for high-photovoltaic-performance Sb_2_(S,Se)_3_ devices. In 2021, Li et al. used hydrothermal technology to deposit Sb_2_(S,Se)_3_ on devices. They achieved a maximum efficiency of 10.7% [11]. Thus, the solution synthesis method is better than the vacuum method. 

Despite the benefits of the hydrothermal method in preparing high-PCE devices, understanding the growth mechanism of the Sb_2_(S,Se)_3_ absorber layer using this technique is challenging. This difficulty primarily arises from the inability to monitor important factors, such as chemical reactions within the hydrothermal solution, temperature, and pH. Furthermore, synthesizing large-scale-area solar cells using this method poses a significant challenge. Therefore, it is urgent to explore a new solution method that involves high-quality preparation, is monitorable, and uses large grain sizes to prepare Sb_2_(S,Se)_3_ devices. CBD technology is an excellent choice for producing Sb_2_(S,Se)_3_ photovoltaic devices, as it offers several advantages over the hydrothermal process. These advantages include (i) the ability to synthesize devices at low temperatures, (ii) the real-time monitoring of precursors to understand the growth mechanism of the absorber layer, (iii) the capability to fabricate devices over large areas, and (iv) the potential for V-shaped band-gap engineering [10]. In 2022, Tang’s group prepared Sb_2_(S,Se)_3_ devices using CBD technology, and the efficiency of the device reached 8.27% [10]. This is the highest PCE achieved for Sb_2_(S,Se)_3_ devices through the CBD method. However, the best PCE of a device using the CBD method is still below the Shockley–Queisser (SQ) limit. Therefore, further optimization of the photovoltaic properties of the devices is needed.

The high-efficiency devices utilized a CdS buffer layer. The cliff-like conduction band offset (CBO) values of the CdS/Sb_2_Se_3_ interface are negative in Sb_2_Se_3_-based devices, producing high recombination losses at the interface [10,12,13]. The Sb_2_(S,Se)_3_ material is similar to Sb_2_Se_3_, and a cliff-like CBO may develop at the interface between CdS and Sb_2_(S,Se)_3_. Thus, the CdS/Sb_2_(S,Se)_3_ interface plays a critical role in determining the photovoltaic properties of Sb_2_(S,Se)_3_ devices. It is essential to optimize the CdS/Sb_2_(S,Se)_3_ interface to enhance the efficiency of an Sb_2_(S,Se)_3_ device. In this study, we introduce the use of a Cd-free buffer layer consisting of Zn_1−x_Sn_x_O_y_ (ZTO) as a replacement for the CdS layer utilizing Afors-het software for the first time. The work is divided into five steps: (I) replacing the CdS buffer layer with the ZTO ternary compound and evaluating its impact on the efficiency of the devices, (II) varying the S content in Sb_2_(S,Se)_3_ and studying its effect on the efficiency, (III) studying the effect of the thickness of Sb_2_(S,Se)_3_ on the efficiency of the device, (IV) investigating the impact of the carrier concentration of ZTO on the PCE of devices, and (V) analyzing the effect of ZTO’s Sn/(Zn+Sn) ratio and thickness on the device’s efficiency. This work presents a novel approach to enhancing the efficiency of devices and serves as a valuable experimental guide. 

## 2. Methods

**Figure 1:**
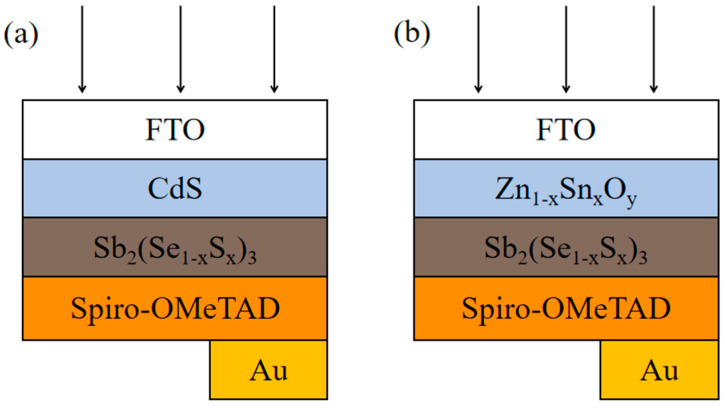
The two structures investigated in this work: (**a**) an Sb_2_(S,Se)_3_ device with a CdS layer and (**b**) an Sb_2_(S,Se)_3_ device with a Zn_1−x_Sn_x_O_y_ layer.

The Afors-het program was used to analyze the impact of the ZTO layer on the efficiency of an Sb_2_(S,Se)_3_ device. The fundamental semiconductor equations employed for the semiconductor device include the Poisson equation, equations for hole and electron current densities, and electron and hole continuity equations:

(1) $$J_{n}=q\mu_{n}n\frac{\partial E_{En}}{\partial x};J_{p}=q\mu_{p}p\frac{\partial E_{Ep}}{\partial x}$$

(2) $$\frac{\partial }{\partial x}(\varepsilon \frac{\partial \phi }{\partial x})=\frac{q}{\varepsilon_{0}}(-n+p-{N_{A}}^{-}+{N_{D}}^{+}+n_{t}+p_{t})$$

(3) $$\frac{\partial n}{\partial t}=G-R_{n}+\mu_{n}E_{x}\frac{\partial n}{\partial x}+D_{n}\frac{\partial^{2}n}{\partial x^{2}};\frac{\partial p}{\partial t}=G-R_{p}+\mu_{p}E_{x}\frac{\partial p}{\partial x}+D_{p}\frac{\partial^{2}p}{\partial x^{2}}$$

where ε and q are the dielectric constant and the electron charge, $N_{A}^{-}$ and $N_{D}^{+}$ are ionized acceptor and donor concentrations, and p and n are hole and electron concentrations. ϕ, $\mu_{p}$, and $\mu_{n}$ are the electrostatic potential, the hole’s mobility, and the electron’s mobility, respectively. $J_{p}$ and $J_{n}$ are the hole’s current density and the electron’s current density. $E_{Fn}$ and $E_{Fp}$ are the electron quasi-Fermi level and hole quasi-Fermi level, respectively. G, $D_{n}$, and $D_{p}$ are the generation rate, the electron’s diffusion coefficient, and the hole’s diffusion coefficient, respectively. $E_{x}$, $R_{n}$, and $R_{P}$ are the electric field, the recombination rate of electrons, and the recombination rate of holes, respectively. Figure 1 displays the simulated device structures with CdS and ZTO electron transport layers. Table 1 and Table 2 list the simulated parameters of the Sb_2_(S,Se)_3_ device with a superstrate structure [14,15,16,17,18,19,20]. 

**Figure 2:**
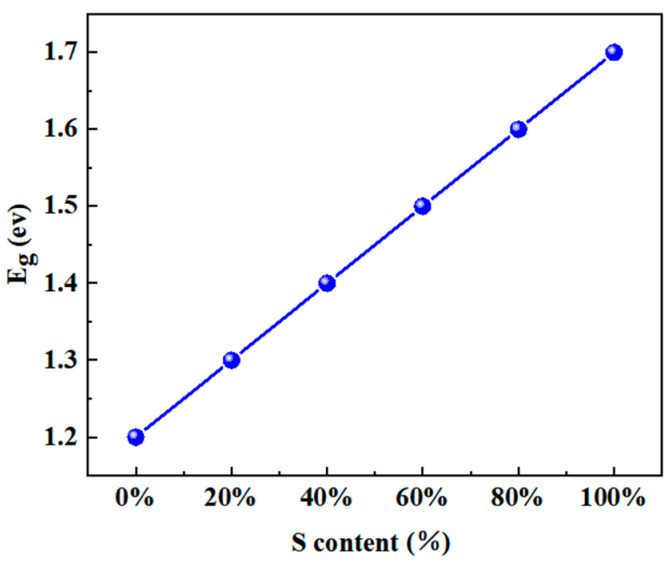
The relationship between the band gap of Sb_2_(S,Se)_3_ and the S content of Sb_2_(S,Se)_3_.

**Figure 3:**
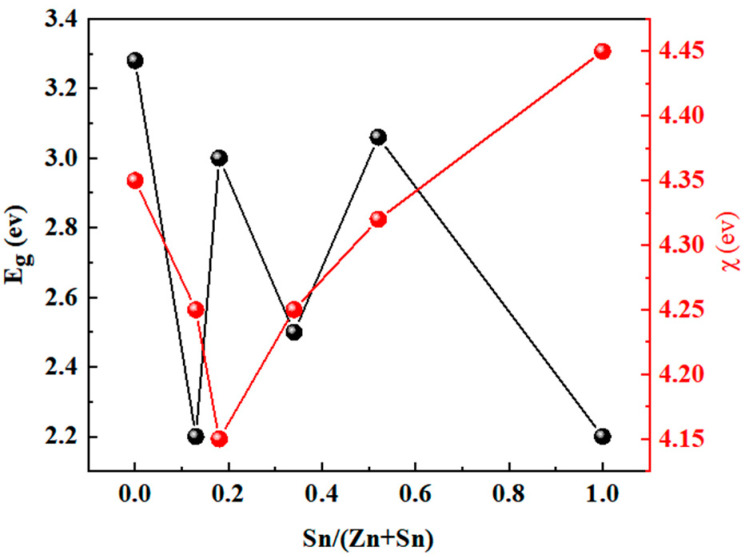
The relationship between the band gap of the ZTO layer, the electron affinity of the ZTO layer, and the Sn/(Zn+Sn) ratio.

To further study the carrier transport between ZTO and Sb_2_(S,Se)_3_, four methods were analyzed. Firstly, the effect of the absorber’s S content on the efficiency of Sb_2_(S,Se)_3_ devices was calculated. Secondly, the impact of the thickness of the Sb_2_(S,Se)_3_ layer on the PCE of the device was investigated. Thirdly, the influence of the ZTO layer’s carrier concentration on the efficiency of the device was examined. Fourthly, the effect of the ZTO layer with varying Sn/(Zn+Sn) ratios on the PCE of the device was analyzed. Lastly, the impact of the thickness of the ZTO layer on the device’s efficiency was investigated. In this study, the band gap of the absorber layer was varied from 1.2 to 1.7 eV to implement the first method. Previous research has shown that the absorber’s band gap decreases linearly with a decrease in the sulfur mole fraction [21]. Figure 2 illustrates the relationship between the S content of the absorber layer and the band gap of the absorber layer, which was used to analyze the effect of the S content on the efficiency of the device. The absorber carrier concentration changes from 1 × 10^15^ cm^−3^ to 1 × 10^21^ cm^−3^. Additionally, Lee et al. suggested that the band gap and electron affinity of the ZTO layer change based on the Sn/(Zn+Sn) ratio [22]. The relationship between the band gap of the ZTO layer, electron affinity, and Sn/(Zn+Sn) ratio is depicted in Figure 3, which was used to analyze the impact of different Sn/(Zn+Sn) ratios on the PCE of solar cells. To study the effect of ZTO thickness on the device’s PCE, the thickness was changed from 10 nm to 100 nm. 

## 3. Results and Discussion

### 3.1. Impact of ZTO Layer 

**Figure 4:**
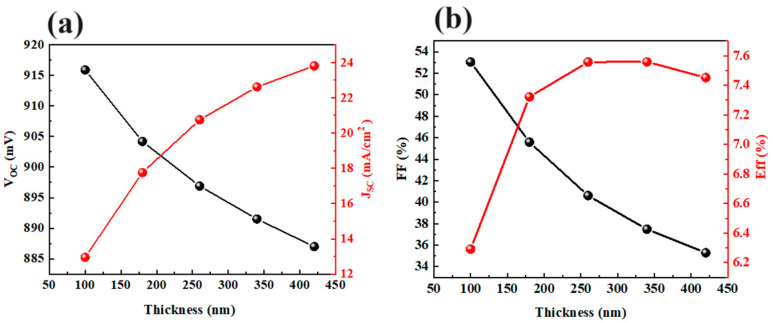
Calculated (**a**) *V_OC_* and *J_SC_* and (**b**) *FF* and efficiency for Sb_2_(S,Se)_3_ devices as a function of the thickness of Sb_2_(S,Se)_3_.

**Figure 5:**
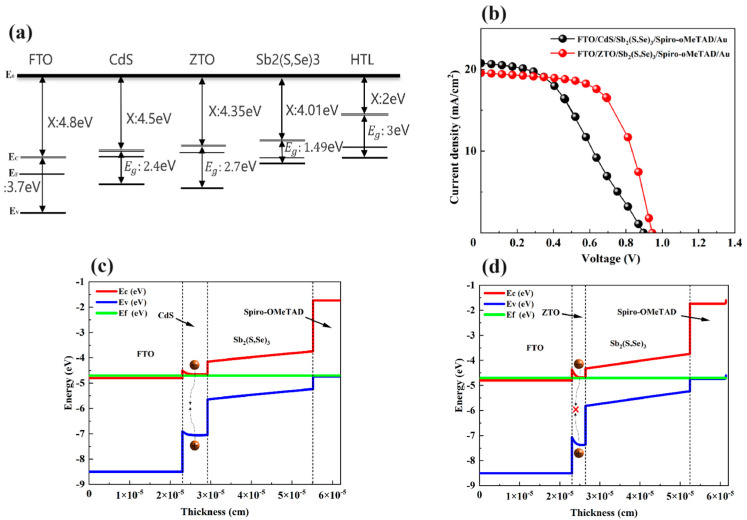
(**a**) The band gap and electron affinity of each layer in Sb_2_(S,Se)_3_ devices, (**b**) *J-V* curves of Sb_2_(S,Se)_3_ devices with CdS and ZTO ETLs, and the energy band diagrams of Sb_2_(S,Se)_3_ devices with (**c**) CdS and (**d**) ZTO layers.

Before investigating the impact of the ZTO layer, it is important to optimize the thickness of the Sb_2_(S,Se)_3_ layer. The thickness of the Sb_2_(S,Se)_3_ layer was varied from 100 nm to 420 nm. The results in Figure 4 demonstrate the influence of the Sb_2_(S,Se)_3_ layer thickness on the photovoltaic parameters of CdS/Sb_2_(S,Se)_3_ solar cells. The thickness of the Sb_2_(S,Se)_3_ layer increases from 100 nm to 420 nm, resulting in a decrease in *V_OC_* from 0.916 V to 0.887 V. However, *J_SC_* increases from 12.95 mA/cm^2^ to 23.81 mA/cm^2^ with the thicker Sb_2_(S,Se)_3_ layer. The FF of the Sb_2_(S,Se)_3_ device decreases from 53.04% to 35.29% as the thickness of the Sb_2_(S,Se)_3_ layer increases. The efficiency of the Sb_2_(S,Se)_3_ device improves from 6.29% to 7.56% when the thickness of the Sb_2_(S,Se)_3_ layer increases from 100 nm to 260 nm, but it then decreases to 7.45% with a further thickness increase. The optimal thickness of the Sb_2_(S,Se)_3_ layer is 260 nm in the CdS/Sb_2_(S,Se)_3_ device. Figure 5 illustrates the comparison of CdS and ZTO layers in Sb_2_(S,Se)_3_ devices as electron transport layers (ETLs) based on the ideal thickness of the Sb_2_(S,Se)_3_ layer. Figure 5a displays the band gap and electron affinity of each layer in the devices. The *J-V* curves of devices with various ETLs are shown in Figure 5b. The device with a CdS layer achieved a PCE of 7.56%, with a *V_OC_* of 0.896 V, a *J_SC_* of 20.75 mA/cm^2^, and an FF of 40.61%. In comparison, the Sb_2_(S,Se)_3_ device with ZTO demonstrated a higher *V_OC_* of 0.942 V, a *J_SC_* of 19.56 mA/cm^2^, an FF of 62.14%, and an efficiency of 11.45%, indicating improved photovoltaic properties. Figure 5c,d depict the energy band diagrams of devices with CdS and ZTO buffer layers. The CBO value at the interface between CdS and Sb_2_(S,Se)_3_ was 0.49 eV, while with ZTO, it decreased to 0.34 eV, suggesting easier electron flow from ZTO to Sb_2_(S,Se)_3_ and reduced carrier recombination (The symbol "X" signifies the decreased carrier recombination). The VBO value at the ZTO/Sb_2_(S,Se)_3_ interface was 1.55 eV, which is higher than the 1.40 eV VBO value at the interface of CdS/Sb_2_(S,Se)_3_. The improved CBO and VBO values at the interface enhance the carrier collection and *V_OC_* of the Sb_2_(S,Se)_3_ device, consistent with the *J-V* results.

### 3.2. Analysis of Photo-Generated Carrier Collection at ZTO/Sb_2_(S,Se)_3_ Interface

#### 3.2.1. Effect of S Content and Thickness in Absorber Layer

**Figure 6:**
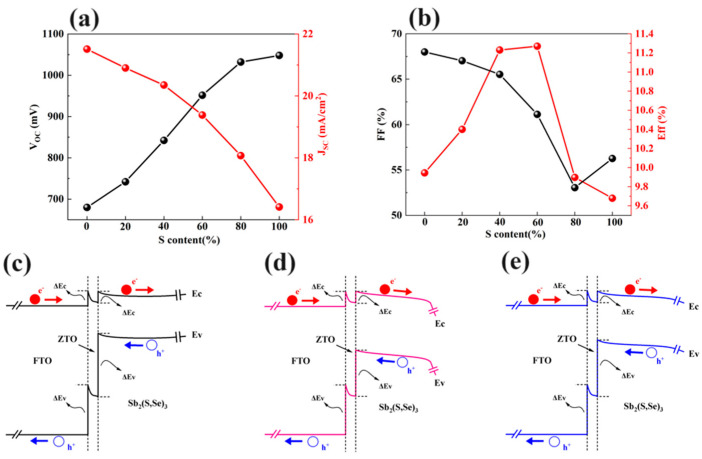
Changes in (**a**) *V_OC_* and *J_SC_* and (**b**) *FF* and efficiency for different S contents in Sb_2_(S,Se)_3_, and the energy band diagrams of FTO/ZTO/Sb_2_(S,Se)_3_ structures with (**c**) 0% S content in the absorber, (**d**) 60% S content in the absorber, and (**e**) 100% S content in the absorber.

To further investigate carrier collection at the ZTO/Sb_2_(S,Se)_3_ interface, the effect of S content in the absorber layer on the photovoltaic performance of the device was studied. Figure 6 illustrates the results of this investigation. As the S content in the absorber layer increases, the *V_OC_* of the device rises from 0.680 to 1.048 V. However, the *J_SC_* of the device decreases from 21.51 to 16.41 mA/cm^2^ with increasing S content, as shown in Figure 6a. Additionally, the *FF* of the device decreases from 68% to 56.26% as the S content increases from 0% to 100% in Figure 6b. Overall, the efficiency of the Sb_2_(S,Se)_3_ device increases with the S content up to 60%, after which it starts to decrease. The energy band diagrams of FTO/ZTO/Sb_2_(S,Se)_3_ with varying S content (0%, 60%, and 100%) are shown in Figure 6c–e. The VBO between the ZTO layer and Sb_2_(S,Se)_3_ layer decreases as the S content increases up to 60%. This lower VBO value indicates a reduced hole barrier at the ZTO/Sb_2_(S,Se)_3_ interface, facilitating the flow of photo-generated holes from the Sb_2_(S,Se)_3_ layer to the ZTO layer and reducing minority carrier recombination at the interface of the ZTO/Sb_2_(S,Se)_3_ heterojunction. Conversely, a higher S content leads to a higher VBO value at the ZTO/Sb_2_(S,Se)_3_ interface, indicating greater difficulty in transporting photo-generated holes from the Sb_2_(S,Se)_3_ absorber to the ZTO layer and resulting in increased carrier recombination. Based on these results, it is recommended that the Sb_2_(S,Se)_3_ absorber layer contain around 60% S content to achieve optimal photovoltaic properties in devices. 

**Figure 7:**
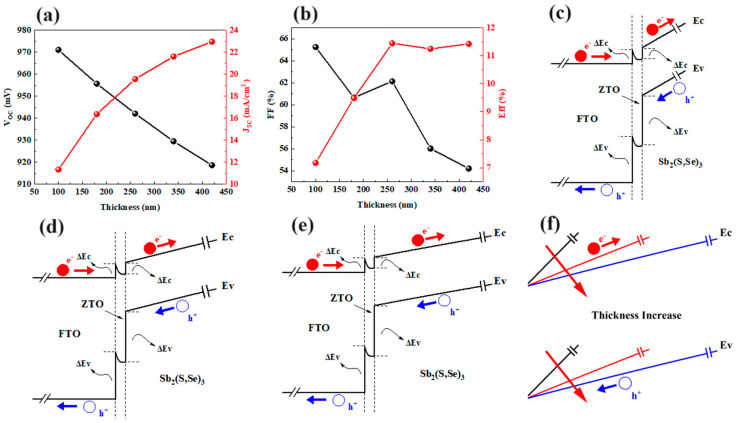
Changes in (**a**) *V_OC_* and *J_SC_* and (**b**) *FF* and efficiency for various thicknesses in Sb_2_(S,Se)_3_; the energy band diagrams of FTO/ZTO/Sb_2_(S,Se)_3_ structures with absorber thicknesses of (**c**) 100nm, (**d**) 260 nm, and (**e**) 420 nm; and (**f**) the effect of the absorber thickness on the built-in potential barrier in the Sb_2_(S,Se)_3_ layer.

Figure 7 displays the effect of Sb_2_(S,Se)_3_ thickness on the photovoltaic parameters of the device. Increasing the thickness of Sb_2_(S,Se)_3_ from 100 nm to 420 nm results in a decrease in *V_OC_* from 0.971 V to 0.919 V, while *J_SC_* increases from 11.33 mA/cm^2^ to 22.96 mA/cm^2^ (Figure 7a). The FF initially decreases from 65.25% to 60.65% as the thickness goes from 100 nm to 180 nm but then slightly increases with further increase in thickness. In Figure 7b, the FF decreases from 62.14% to 54.2% as the thickness increases from 260 nm to 420 nm. Overall, the efficiency of the Sb_2_(S,Se)_3_ devices increases from 7.18% to 11.45% as the absorber layer thickness ranges from 100 nm to 260 nm. Further increases in thickness have a minimal impact on device efficiency. The energy band diagrams of FTO/ZTO/Sb_2_(S,Se)_3_ with various thicknesses of 100 nm, 260 nm, and 420 nm are illustrated in Figure 7c–e. The built-in potential barrier in the Sb_2_(S,Se)_3_ layer decreases as the absorber layer’s thickness increases from 100 nm to 260 nm (Figure 7f). This lower built-in potential barrier improves the transport of photo-generated electrons from the ZTO/Sb_2_(S,Se)_3_ interface to the Sb_2_(S,Se)_3_ layer, as well as the collection of photo-generated holes from the Sb_2_(S,Se)_3_ layer to the ZTO/Sb_2_(S,Se)_3_ interface. A thickness of 260 nm results in reduced carrier recombination in the Sb_2_(S,Se)_3_ layer and enhances the device’s efficiency. With further increases in thickness, the reduction in the built-in potential barrier in Sb_2_(S,Se)_3_ becomes slower, potentially leading to similar transport of photo-generated carriers in the Sb_2_(S,Se)_3_ layers with various thicknesses of 260 nm and 420 nm. Consequently, the efficiency of the device remains relatively constant with increasing thickness. The optimal thickness of Sb_2_(S,Se)_3_ is 260 nm in the device.

#### 3.2.2. Impact of Carrier Concentration of ZTO Layer

**Figure 8:**
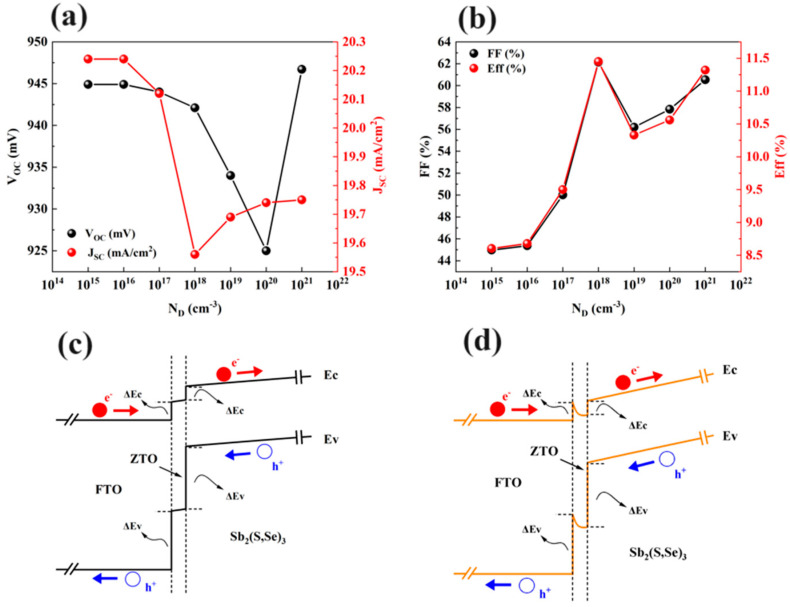
Calculated (**a**) *V_OC_* and *J_SC_* and (**b**) *FF* and efficiency for Sb_2_(S,Se)_3_ devices as a function of the carrier concentration of ZTO, and the energy band diagrams of FTO/ZTO/Sb_2_(S,Se)_3_ structures when ZTO’s carrier concentration is (**c**) 1 × 10^15^ cm^−3^ and (**d**) 1 × 10^18^ cm^−3^.

Figure 8 displays the impact of the carrier concentration (*N_D_*) of the ZTO layer on the four parameters of the Sb_2_(S,Se)_3_ device. As the carrier concentration of the ZTO layer changes to 1 × 10^20^ cm^−3^, the *V_OC_* of the device is reduced from 0.945 V to 0.925 V. Further increasing the carrier concentration results in the *V_OC_* of the device changing from 0.925 V to 0.947 V. A study indicated that the enhanced built-in electric field at the interface between the ETL and absorber layer leads to an improvement in the *V_OC_* of the device [23]. A ZTO layer with a high carrier concentration of 1 × 10^21^ cm^−3^ can create an enhanced built-in electric field at the interface of the ZTO/Sb_2_(S,Se)_3_ heterojunction. This enhanced built-in electric field plays a crucial role in raising the device’s *V_OC_* from 0.925 V to 0.947 V. The *J_SC_* of the device varies from 20.24 mA/cm^2^ to 19.56 mA/cm^2^ as the carrier concentration of the ZTO layer changes from 1 × 10^15^ cm^−3^ to 1 × 10^18^ cm^−3^. *J_SC_* then increases as *N_D_* increases from 1 × 10^18^ cm^−3^ to 1 × 10^21^ cm^−3^. These numerical results are shown in Figure 8a. Figure 8b shows that the *FF* and *PCE* of the device increase as ZTO’s *N_D_* increases from 1 × 10^15^ cm^−3^ to 1 × 10^18^ cm^−3^. However, with a further increase in carrier concentration from 1 × 10^18^ cm^−3^ to 1 × 10^19^ cm^−3^, the FF and PCE decrease. Finally, as *N_D_* changes from 1 × 10^19^ cm^−3^ to 1 × 10^21^ cm^−3^, the FF and PCE of the device increase once again. Figure 8c,d illustrate the detailed energy band diagrams of FTO/ZTO/Sb_2_(S,Se)_3_. The CBO value and VBO value are 0.34 eV and 1.55 eV when the *N_D_* of ZTO is 1 × 10^15^ cm^−3^ in Figure 8c. A hole barrier can be seen at the interface between FTO and ZTO, hindering the collection of photo-generated holes from ZTO to the FTO layer. However, the CBO value and VBO value of the ZTO/Sb_2_(S,Se)_3_ interface stay at 0.34 eV and 1.55 eV in Figure 8d when the carrier concentration of ZTO further increases to 1 × 10^18^ cm^−3^. The disappearance of the hole barrier at the FTO/ZTO interface enhances the flow of photo-generated holes from ZTO to FTO, reducing the recombination of minority carriers and improving the *FF* of the Sb_2_(S,Se)_3_ device. Therefore, to achieve the best efficiency of the device, the ZTO layer should have an *N_D_* of 1 × 10^18^ cm^−3^. 

#### 3.2.3. Effect of Sn/(Zn+Sn) Ratio in ZTO

**Figure 9:**
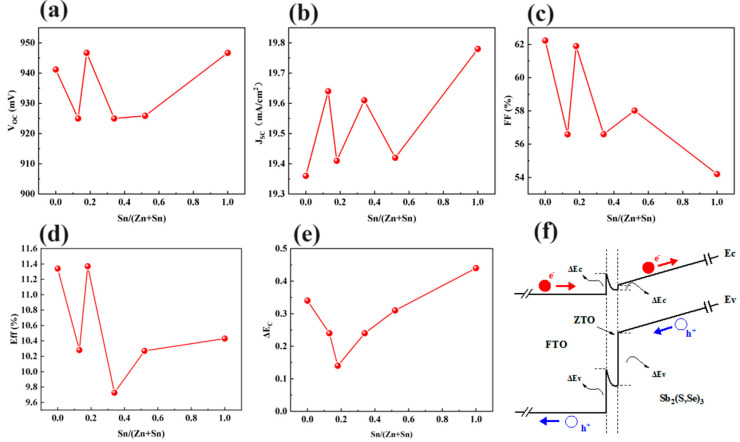
The calculated (**a**) *V_OC_*, (**b**) *J_SC_*, (**c**) *FF*, and (**d**) efficiency of the Sb_2_(S,Se)_3_ device as a function of the Sn/(Zn+Sn) ratio of ZTO, (**e**) the effect of the Sn/(Zn+Sn) ratio of ZTO on the conduction band offset (CBO), and (**f**) the energy band diagram of the FTO/ZTO/Sb_2_(S,Se)_3_ structure (the Sn/(Zn+Sn) ratio of ZTO is 0.18).

In order to analyze the effect of the Sn/(Zn+Sn) ratio on the photo-generated minority carrier collection properties of Sb_2_(S,Se)_3_ devices, the Sn/(Zn+Sn) ratio in ZTO was varied from 0 to 1. In Figure 9a–d, the impact of the Sn/(Zn+Sn) ratio on the photovoltaic parameters of the device is illustrated. The optimal Sn/(Zn+Sn) ratio is found to be 0.18, resulting in the highest *V_OC_* for the device. Additionally, the optimal *J_SC_* of 19.76 mA/cm^2^ is achieved with a Sn/(Zn+Sn) ratio of 1.0. The effect of the Sn/(Zn+Sn) ratio on the *FF* of the devices is also studied, with the best *FF* obtained when the ZTO layer had a Sn/(Zn+Sn) ratio of 0.18. Furthermore, the PCE of the device is studied in relation to the Sn/(Zn+Sn) ratio. The best PCE is observed when the ZTO layer has a Sn/(Zn+Sn) ratio of 0.18. These results indicate that ZTO with a Sn/(Zn+Sn) ratio of 0.18 enhances the *V_OC_*, *FF*, and PCE of the device. To further investigate the impact of the Sn/(Zn+Sn) ratio on the minority carrier transport at the ZTO/Sb_2_(S,Se)_3_ interface, the simulation results are analyzed in Figure 9e,f. The effect of different Sn/(Zn+Sn) ratios on the CBO value at the interface of ZTO/Sb_2_(S,Se)_3_ is shown in Figure 9e. It is observed that the CBO value decreases from 0.34 eV to 0.13 eV as the Sn/(Zn+Sn) ratio increases from 0 to 0.18. Subsequently, the CBO value is enhanced from 0.13 eV to 0.43 eV as the Sn/(Zn+Sn) ratio increases from 0.18 to 1. The energy band diagram of FTO/ZTO (with a Sn/(Zn+Sn) ratio of 0.18)/Sb_2_(S,Se)_3_ is shown in Figure 9f. This ratio results in a lower conduction band offset (CBO) value at the interface between ZTO and Sb_2_(S,Se)_3_. Previous research has shown that a lower CBO value at the interface between the electron transport layer and the absorber layer can reduce interface recombination and the open-circuit voltage deficit and ultimately improve the efficiency of devices [24]. Therefore, the decreased CBO at the ZTO/Sb_2_(S,Se)_3_ interface enhances the *V_OC_* and efficiency of the Sb_2_(S,Se)_3_ device.

#### 3.2.4. Effect of ZTO Thickness

**Figure 10:**
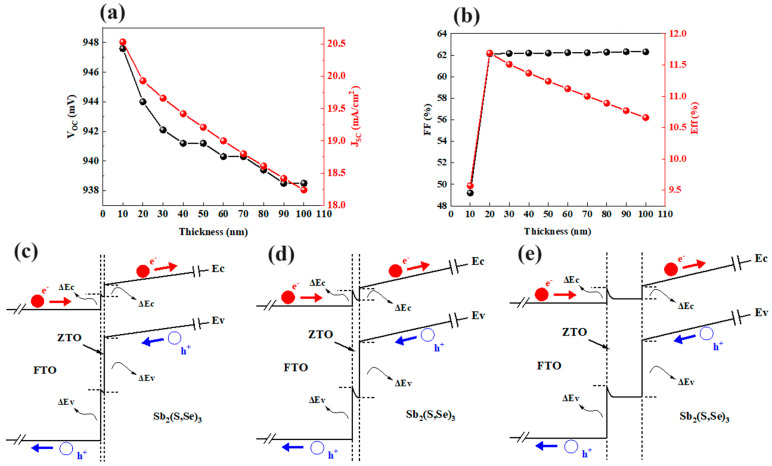
Changes in (**a**) *V_OC_* and *J_SC_* and (**b**) *FF* and efficiency for different thicknesses of the ZTO layer, and the energy band diagrams of FTO/ZTO/Sb_2_(S,Se)_3_ structures with ZTO thicknesses of (**c**) 10 nm, (**d**) 20 nm, and (**e**) 100 nm.

We investigate the effect of ZTO thickness on the photovoltaic parameters of the device in Figure 10. The dependence of *V_OC_* on the ZTO thickness is illustrated in Figure 10a. It is observed that the *V_OC_* of the device generally decreases as the ZTO thickness increases from 10 nm to 100 nm. Similarly, *J_SC_* decreases as the ZTO thickness increases. Figure 10b depicts the effect of ZTO thickness on the FF and efficiency of the device. The FF increases with the thickness of the ZTO layer up to 20 nm but then plateaus with further increases in thickness. The efficiency of the device increases with the ZTO thickness up to 20 nm but then decreases with further increases in thickness. These results reveal that the optimal ZTO thickness is 20 nm. Figure 10c–e display the energy band diagrams of FTO/ZTO/Sb_2_(S,Se)_3_ structures with various ZTO thicknesses. The CBO and VBO values at the interface of the ZTO/Sb_2_(S,Se)_3_ heterojunction stay at 0.34 eV and 1.55 eV as the thickness of ZTO increases. The built-in electric field from ZTO to FTO can be seen at the FTO/ZTO interface. An enhanced built-in electric field is observed at the FTO/ZTO interface with increasing ZTO thickness up to 20 nm, facilitating the flow of photo-generated holes from the ZTO layer to the FTO layer. This reduction in carrier recombination in the ZTO layer is essential for improving efficiency. However, when the ZTO thickness reaches 100 nm, a flat band is seen at the CBM and VBM, which hinders the carrier flow and leads to increased carrier recombination in the ZTO layer, ultimately limiting the device’s efficiency. In conclusion, a ZTO thickness of 20 nm is optimal for achieving the highest efficiency of the device.

## 4. Conclusions

This study presents a theoretical simulation comparing the use of ZTO and CdS as ETLs in Sb_2_(S,Se)_3_ devices. The results show that the CBO at the ZTO/Sb_2_(S,Se)_3_ interface is cliff-like but has a lower value of 0.34 eV compared to the interface of CdS/Sb_2_(S,Se)_3_. The lower CBO value limits the recombination of minority carriers at the interface of ZTO/Sb_2_(S,Se)_3_. Additionally, the VBO value at the interface of ZTO/Sb_2_(S,Se)_3_ is higher than that at the CdS/Sb_2_(S,Se)_3_ interface. These improved CBO and VBO values at the ZTO/Sb_2_(S,Se)_3_ interface enhance the transport of carriers, resulting in an increase in the PCE of the devices from 7.56% to 11.45%. The presence of 60% S content in the Sb_2_(S,Se)_3_ layer constructs a low hole barrier at the interface of the ZTO/Sb_2_(S,Se)_3_ heterojunction, reducing photo-generated hole losses at the interface and leading to improved photovoltaic performance. The absorber with a thickness of 260 nm has a lower built-in potential barrier, reducing the carrier recombination in the Sb_2_(S,Se)_3_ absorber and improving the efficiency of the device. Furthermore, the hole barrier disappears at the interface of FTO/ZTO when the ZTO layer has a carrier concentration of 1 × 10^18^ cm^−3^, promoting the PCE of the devices. A Sn/(Zn+Sn) ratio of 0.18 in the ZTO layer optimizes the CBO value at the ZTO/Sb_2_(S,Se)_3_ interface, reducing carrier recombination at the interface. Moreover, a ZTO layer with a thickness of 20 nm produces an enhanced built-in electric field at the FTO/ZTO interface, further optimizing the efficiency of the devices. 

## Figures and Tables

**Table 1:** Simulated parameters of the solar cells.

Parameters	FTO	Sb_2_(S,Se)_3_	Spiro-oMeTAD	CdS	ZTO
Thickness (nm)	230	260	90	62	34
ε_r_	9	14.38	3	10	9
χ (eV)	4.8	4.01	2	4.5	4.35
E_g_ (eV)	3.7	1.49	3	2.4	2.7
N_C_ (cm^−3^)	2.2 × 10^18^	2.2 × 10^18^	2.5 × 10^18^	2.2 × 10^18^	2.2 × 10^18^
N_V_ (cm^−3^)	1.8 × 10^19^	1.8 × 10^20^	1.8 × 10^19^	1.8 × 10^19^	1.8 × 10^19^
µ_e_ (cm^2^/V^.^s)	20	14	1.0 × 10^−4^	100	30
µ_h_ (cm^2^/V^.^s)	10	2.6	2.0 × 10^−4^	25	5
N_A_ (cm^−3^)	–	1.0 × 10^14^	5.0 × 10^18^	–	10
N_D_ (cm^−3^)	1.0 × 10^20^	–	–	4.0 × 10^17^	10^15^–10^21^
V_th,e_ (cm/s)	1.0 × 10^7^	1.0 × 10^7^	1.0 × 10^18^	1.0 × 10^7^	1.0 × 10^7^
V_th,p_ (cm/s)	1.0 × 10^7^	1.0 × 10^7^	1.0 × 10^18^	1.0 × 10^7^	1.0 × 10^7^

**Table 2:** Bulk defect parameters of the solar cells.

	FTO	Sb_2_(S,Se)_3_		Spiro-oMeTAD	CdS	ZTO
		Defect 1	Defect 2			
Defect Type	Single Acceptor	Single Acceptor	Single Acceptor	Single Acceptor	Single Donor	Single Neutral
N_t_	1.0 × 10^15^	1.3 × 10^14^	1.0 × 10^15^	1.0 × 10^18^	1.0 × 10^14^	3.0 × 10^17^
σ_e_(cm^2^)	1.0 × 10^−15^	1.99 × 10^−14^	1.99 × 10^−14^	1.0 × 10^−15^	3.0 × 10^−15^	1.0 × 10^−14^
σ_h_(cm^2^)	1.0 × 10^−15^	1.99 × 10^−14^	1.99 × 10^−14^	1.0 × 10^−15^	2.0 × 10^−14^	1.0 × 10^−15^

## Data Availability

The raw data supporting the conclusions of this article will be made available by the authors on request.
